# Drug Encapsulated Lipid-Polymeric Nanohybrid as a Chemo-therapeutic Platform of Cancer

**DOI:** 10.7150/ntno.81173

**Published:** 2023-01-16

**Authors:** Rahul Kumar, Vinish Ranjan Srivastava, Supratim Mahapatra, Daphika S Dkhar, Rohini Kumari, Kumari Prerna, Vikash Kumar Dubey, Pranjal Chandra

**Affiliations:** School of Biochemical Engineering, Indian Institute of Technology (BHU), Varanasi- 221005, Uttar Pradesh, India.

**Keywords:** nanohybrid system, theranostics, methotrexate, lipid, drug delivery, MCF-7 cells

## Abstract

The focus of this research is to design a bioengineered drug delivery vehicle that is efficient in anti-cancer drug delivery in a controlled manner. The experimental work focuses on constructing a methotrexate-loaded nano lipid polymer system (MTX-NLPHS) that can transport methotrexate (MTX) in MCF-7 cell lines in a controlled manner through endocytosis via phosphatidylcholine. In this experiment, MTX is embedded with polylactic-co-glycolic acid (PLGA) in phosphatidylcholine, which acts as a liposomal framework for regulated drug delivery. Scanning electron microscopy (SEM), Fourier transform infrared spectroscopy (FTIR), X-ray diffraction (XRD), and dynamic light scattering (DLS) were utilized to characterize the developed nanohybrid system. The particle size and encapsulation efficiency of the MTX-NLPHS were found to be 198 ± 8.44 nm and 86.48 ± 0.31 %, respectively, which is suitable for biological applications. The polydispersity index (PDI) and zeta potential of the final system were found to be 0.134 ± 0.048 and -28 ± 3.50 mV, respectively. The lower value of PDI showed the homogenous nature of the particle size, whereas higher negative zeta potential prevented the system from agglomeration. An *in vitro* release kinetics was conducted to see the release pattern of the system, which took 250 h for 100% drug release This kind of system may carry the drug for a long time in the circulatory system and prevent the drug discharge. Other cell culture assays such as 3-(4, 5-dimethyl thiazolyl-2)-2, 5-diphenyltetrazolium bromide (MTT) and reactive oxygen species (ROS) monitoring were used to see the effect of inducers on the cellular system. MTT assay showed cell toxicity of MTX-NLPHS reduced at the lower concentration of the MTX, however, toxicity increased at the higher concentration of the MTX as compared to free MTX. ROS monitoring c revealed more scavenging of ROS using MTX-NLPHS as compared to free MTX. Confocal microscopy suggested the MTX-NLPHS induced more nuclear elongation with cell shrinkage comparatively.

## Introduction

Nanocarrier, a colloidal system, has been widely investigated in therapeutic directions that is smaller than 500 nm in size 1,2. Nanocarriers may change the basic properties and drug bioactivity due to their high surface area 3. Nanocarriers may enable drug delivery systems with improved stability, solubility, site-specificity, and tailored drug release 4,5. These are also used widely because of their inactive nature and deemed to be a safe medium 6. These nanomaterials will circulate for a long time in the circulatory system and will constantly release drugs, circumventing the endosome-lysosomal pathway 7. Furthermore, the physicochemical characteristics of nanocarriers may be altered by changing their compositions, sizes, shapes, and surface chemistry 8. The ultimate objective of employing nanocarriers in pharmaceutical delivery is to cure an ailment effectively while minimizing negative effects 9,10. The optimum drug conjugated nanosystem is selected based on the drug encapsulation efficiency and biochemical features of the targeted medications chosen for therapy 11. However, nanocarrier toxicity must be addressed when contemplating the application of nanomedicine 12. Biodegradable polymeric nanoparticles and liposomes are the two emerging popular categories of drug nanocarriers, as indicated by a growing number of approved drug products, research reports, and cumulative numbers of clinical trials 13. The two classes have virtues and detriments relating to biological and physiochemical features. In the past, innumerable types of lipids have been used in many drug conveyance methods including solid lipid nanoparticles, liposomes, drug-lipid complexes, and nanostructured lipid carriers 14,15. As long as the lipid components of the liposome are obtained from natural sources, they are biodegradable, biocompatible, flexible, mildly toxic or nontoxic, and non-immunogenic for both systemic and non-systemic use 16.

Liposomal drugs have some disadvantages related to chemical and physical stability, sterilization, batch-to-batch reproducibility, manufacturing scale-up as well as drug entrapment 17. However, polymeric nanoparticles have merits in terms of a larger variety of preparation methodologies, the ability of tissue penetration, smaller particle size, improved stability in biological fluids, availability of various polymers, release profiles, and versatile drug loading 18. The use of toxic organic solvents in the manufacturing process, drug leakage before reaching target tissues, poor drug encapsulation for hydrophilic drugs, scale-up issues, polymer cytotoxicity, and polymer degradation are all disadvantages of polymeric nanoparticles 19. Therefore, in this formulation, we tried to improve the physiochemical and pharmacokinetic parameters of the nanocarriers in terms of size, shape, stability, high drug loading efficiency, shelf-life, and controlled drug release pattern by merging lipids with drug-encapsulated PLGA and selecting optimized formulation parameters. Polymer cores in combination with lipids bilayer provide a theoretically superior delivery system which is called nano lipid-polymer hybrid system (NLPHS) 20,21. The NLPHS is submicron-sized solid particles made up of lipids and polymers. The hybrid system can adsorb, entrap, or covalently link a variety of bioactive molecules, such as genes, drugs, targeting ligands, and proteins 22. Polycaprolactone (PCL), PLGA, and albumin are being widely used as polymeric carrier materials because of their biodegradability, nontoxicity, biocompatibility, and subsequent use in approved products 23. Anionic, cationic, zwitterionic, and neutral polymeric carrier materials are being widely investigated for the synthesis of nano lipid systems (NLS) 24. In the present investigation, we have synthesized a controlled and efficient drug delivery system which may overcome the problem of premature drug elimination, non-specific binding, reduced toxicity, and improved half-life of drugs while circulating in the reticuloendothelial system. MTX, a possible anti-cancer medication, has been integrated with the biodegradable PLGA in the formulations' core. Furthermore, the system's PLGA core was encased in a shell made of phosphatidylcholine. It is conceivable that the MTX-loaded NLPHS (MTX-NLPHS) may combine the essential properties of PLGA and lipids for the construction of a potent theranostics module. However, NLS was also considered in this investigation for comparative study.

## Materials and Methods

Phosphatidylcholine (PC, Product No. Y0001950), PLGA (50:50, Product No. P2191), MTX (≥98%, Product No. M8407), 2',7'-dichlorofluorescein diacetate dye (DCFDA, ≥95%, Product No. 35845), 4',6-diamidino-2-phenylindole (DAPI, ≥98%, Product No. D9542), and propidium iodide (PI, ≥94%, Product No. 81845) were obtained from Sigma Aldrich, USA. MCF-7 (human breast cancer cell line) was bought from National Centre for Cell Sciences (NCCS), Pune, India. Moreover, trypsin-ethylenediamine tetra acetic acid (EDTA, Molecular Biology Grade, Product No. 324503), chloroform (≥ 99 %, Product No. 107024), dimethyl sulfoxide (≥ 99.9 %, DMSO, Product No. 102952) were purchased from Merck Millipore, USA. The remaining solvents and reagents were either analytical- or HPLC-grade purchased from SRL, India.

### Synthesis of nano lipid system (NLS) and nano lipid polymer hybrid system (NLPHS) for anti-cancer drug delivery

The MTX-NLS and MTX-NLPHS were synthesized using a single emulsion solvent evaporation method 25 and a one-step precipitation process 26, respectively. In the MTX-NLPHS, the aqueous phase was prepared by dissolving 5% dextrose in milli-Q-water. After that, the aqueous phase was placed inside a serological water bath at 60°C for 15 min before being placed on a magnetic stirrer at 25°C and 800 rpm. The aqueous phase contains 30% phosphatidylcholine by weight of total PLGA. Then, 30 mg of PLGA along with 10 mg of MTX were added to 5 mL of chloroform to form an organic phase. The organic phase is added to the aqueous phase placed on a magnetic stirrer at 60°C and 200rpm. After that, the solution was allowed for sonication at 40% amplitude for 2 min. Further, the sample was centrifuged under cold conditions for 12 min at 12,000 rpm to separate the nano-formulations. To separate unbound chemicals, the formulations were cleaned two times with Milli Q water. The cleaned sample was re-dissolved in a sucrose solution at a 2% (w/v) concentration to prevent the formulation from freezing. The sample was then powdered and kept in a vacuum desiccator for further use.

### Dynamic light scattering

The diameter, zeta potential, polydispersity index (PDI), of MTX-NLS and MTX-NLPHS in an aqueous solution was determined by DLS (Zeta Nano S Red badge, Malvern Panalytical Ltd. Worcestershire, United Kingdom). In a brief, each sample was diluted fifteen times with organic solvent. The diluted solution (100 μg/mL) was then kept in a sonicator for 2 min at 30% amplitude. The cold condition was maintained throughout the sonication. The sonicated sample was collected in polystyrene cuvette at room temperature, and the particle size was measured at a constant 90° angle.

### Scanning electron microscopy

To investigate the morphological characteristics of MTX-NLS and MTX-NLPHS, SEM (Evo-Scanning Electron microscope MA15/18) was utilized. The reference voltage on the SEM was 26 kV. Briefly, 1 mg of lyophilized sample was dissolved in 5 mL of solvent. Subsequently, sonication was allowed under cold conditions to separate the particles. 12 μL of the sample was placed on a rectangular coverslip and separately dried under reduced pressure. The sample was coated with gold to enable conductivity. Before the completion of the scanning, the conductive sample was held on a copper stub.

### Fourier-transform infrared spectroscopy

The IR spectra of MTX-NLS and MTX-NLPHS were analyzed using IR (Nicolet iS5, Thermo Electron Scientific Instruments LLC, Madison, U.S.A) to validate the various functionalities ((OH, CH_2,_ PO_4_^3-^, NH_2_, NH-CH_2_)) intact with them. A hydraulic press (HP-15-TM; HP-mini) producing 15 tons of force was used to make 13 mm pellets. The dried samples (1% by weight) of MTX-NLS and MTX-NLPHS were mixed individually with the potassium bromide (KBr) while making the pellets. Following that, the obtained pellets were scanned and data was captured from wavenumber 4000 cm^-1^ to 500 cm^-1^.

### X-ray diffraction

To observe the crystallinity of MTX-NLS and MTX-NLPHS, XRD was conducted. The XRD pattern was observed using a Cu Kα radiation source with a potential of 30 kV. The diffractogram was produced by X-ray scanning from a beginning angle of 2θ = 5° to 2θ = 20° at a scanning speed of 0.03° min^-1^. All of the parameters were kept at room temperature while experimenting.

### *In vitro* release kinetics

To quantify the amount of drug released following lipid and polymer disintegration*, in vitro* drug release investigation was conducted. At physiological pH, 5 mg of MTX-NLPHS was added to 3 mL of PBS. The suspension was then placed into the dialysis tube with a 10,000 Da molecular cut-off. The dialysis tube was put horizontally in a sterile beaker containing 100 mL of PBS, and the system was kept at 37 °C and 600 rpm for 24 h. A 1 mL sample was taken and replaced with the same amount of PBS at regular intervals. The obtained sample was kept in a refrigerator and the amount of drugs released was quantified using the UV-spectrophotometer.

### 3-(4,5-dimethylthiazol-2-yl)-2,5-diphenyltetrazolium bromide (MTT Assay)

*In vitro* study included culturing of human breast cancer cells MCF-7. These cells were cultured in Dulbecco's Modified Eagle Medium (DMEM) (product no.- 11995-065, ThermoFisher Scientific) supplemented with 10% fetal bovine serum (FBS) (product no.- RM9955, HiMedia), 100 U/mL penicillin/streptomycin (product no.- A018, HiMedia). The cells were maintained in a humidified incubator at 37°C in an atmosphere of 5% CO2.

MCF-7 cells were seeded in triplicate in a 96-well plate at a density of 10000 cells/well. Following overnight adherence, the media was replaced with a fresh medium containing treatments comprising MTX-NLS and MTX-NLPHS in five independent groups to see their comparative effects on MCF-7 cells. In each group, MTX concentrations varies from 20 μg/mL to 100 μg/mL. For all of the tests, the treated cells were incubated for 48 h. After incubation, media was decanted and the cells were then rinsed thrice with nuclease free water to discrete the remaining amounts of formulations. The cells were subsequently incubated with 20 μL of freshly prepared MTT (5 mg/mL) in PBS at 37°C for 4 h. After 4 h, the supernatant was removed and the formed formazan crystals were then dissolved using 100 μL of DMSO, and the cell viability was determined by measuring absorbance at 595 nm using an ELISA plate reader.

### Reactive oxygen species (ROS) monitoring

This investigation was conducted for real-time monitoring of ROS in MCF-7 cells using cell permeable ROS sensor dye 2',7'-dichlorofluorescein diacetate (DCFDA). After this, cells were taken in a 6-well plate with each well having 1×10^4^ cells. Further, cells were induced with 50 μM H_2_O_2_ solution (as a positive control), free MTX, and MTX-NLPHS for 48 h, however, cells without treatment were taken as a negative control. The experiments were performed in triplicate. After 48 h of incubation, cells were washed with 1X PBS and further incubated with 10 μM of DCFDA dye for 1 h and further fixed using 4% paraformaldehyde on the glass slides to observe changes with confocal microscopy. The interaction of DCFDA dye with intracellular ROS produces green fluorescence, which can be measured at excitation/emission spectra of 485/535 nm.

### Morphological study of MCF-7 cell lines induced with MTX and MTX-NLPHS

To analyze the morphology of MCF-7 cell lines, DAPI and PI dyes were used. DAPI is a blue-fluorescent producing nuclear staining dye that binds to the minor groove of DNA by permeating the cell membrane whereas PI is a red-fluorescent producing nuclear dye that can only permeate dead cells 27. To observe nuclear morphology in MCF-7 cell lines, cells were seeded at a density of 1×10^4^ cells per well in a 6-well plate and incubated overnight for cell adherence. Further, cells were treated with MTX and MTX-NLPHS and incubated for 48 h. Further, the treated cells were rinsed with PBS and then fixed with 4% paraformaldehyde for 15 min at 4 °C. The cells were again rinsed with PBS and then incubated with 1 μg/mL DAPI and 2 μg/mL PI in the designated wells for 30 min and 5 min respectively in the dark at RT. After incubation, cells were rinsed with PBS and then visualized under confocal microscope using excitation/emission spectra of 358/461 nm and 535/617 nm for DAPI and PI respectively. The images were taken at 20X magnification.

## Results and Discussions

### Encapsulation efficiency/Particle size/Polydispersity/Zeta potential

**Figure 2:**
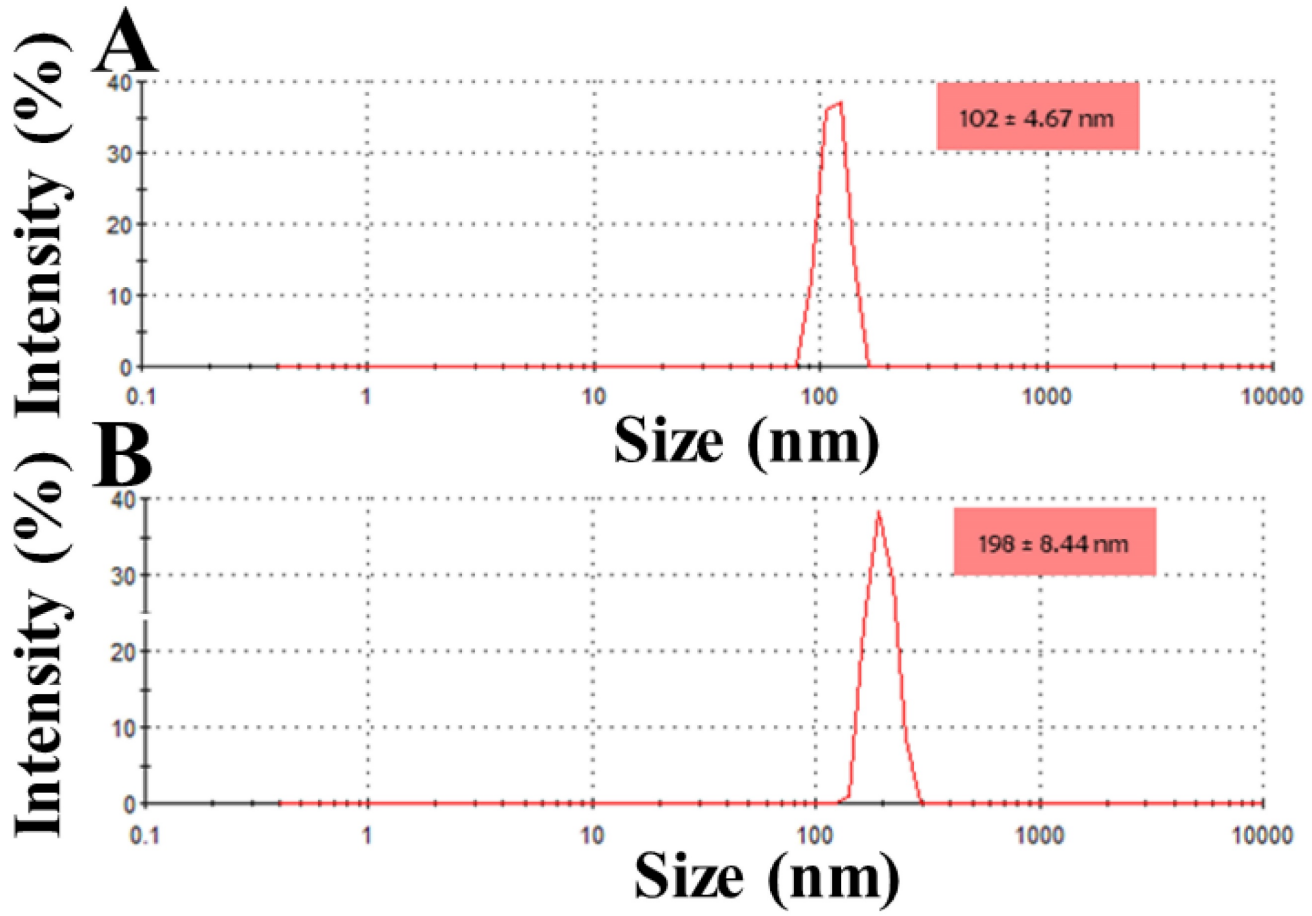
Shows the DLS pattern with size distribution (nm) for (A) MTX-NLS and, (B) MTX-NLPHS.

The encapsulation efficiency of the formulated system is one of the relevant criteria to determine its application. The percentage of drugs entrapped in MTX-NLS and MTX-NLPHS was found to be 79.40 ± 0.28% and 86.48 ± 0.31%, respectively **(Table 1).** The relatively higher encapsulation efficiency in percentage is found due to the PLGA matrix in the core of the hybrid system**.** Results can be interpreted that when the diameter of the carrier grows, the effectiveness of the drug encapsulation increases**.** A nanoparticle analyzer was used to determine the average diameter of MTX-NLS and MTX-NLPHS. The particle size distribution of MTX-NLS and MTX-NLPHS was found to be in the range of 100-200 nm **(Table 1) (Fig. 2)**. The relatively larger size of the MTX-NLPHS is observed due to the blending of PLGA with lipid molecules. An essential parameter that provides distribution of particle size is the PDI. The PDI value of MTX-NLS and MTX-NLPHS was found to be less than 0.2, which means the particle size distribution is likely to be uniform. The above findings are also comparable with previous studies, where particle sizes were distributed equally 28. Zeta potential is a critical indicator of stability, biological activity, and cellular interactions in the medium. Strong repulsive interactions between the carriers are facilitated by the overall high value of zeta potential, whether negative or positive, avoiding particle agglomeration 19. The negative charge on the surface of MTX-NLS and MTX-NLPHS is indicated by the zeta potential as shown in **Table 1**. The overall surface charge is obtained because of the phosphate group of the lipid. The reduction of the negative charge on the surface of MTX-NLPHS as compared to MTX-NLS is due to the phosphate group may interact with PLGA molecules through various interactions in the system. Furthermore, the sizes reported by the zeta sizer differ from the sizes obtained by SEM. The zeta sizer examines average particle size while the SEM governs individual particle size, which could account for the size discrepancy. Hydrodynamic diameters of the system obtained are in the nanoscale range, making it appropriate for a wide range of biological applications.

### Morphological characterization of the hybrid system

**Figure 3:**
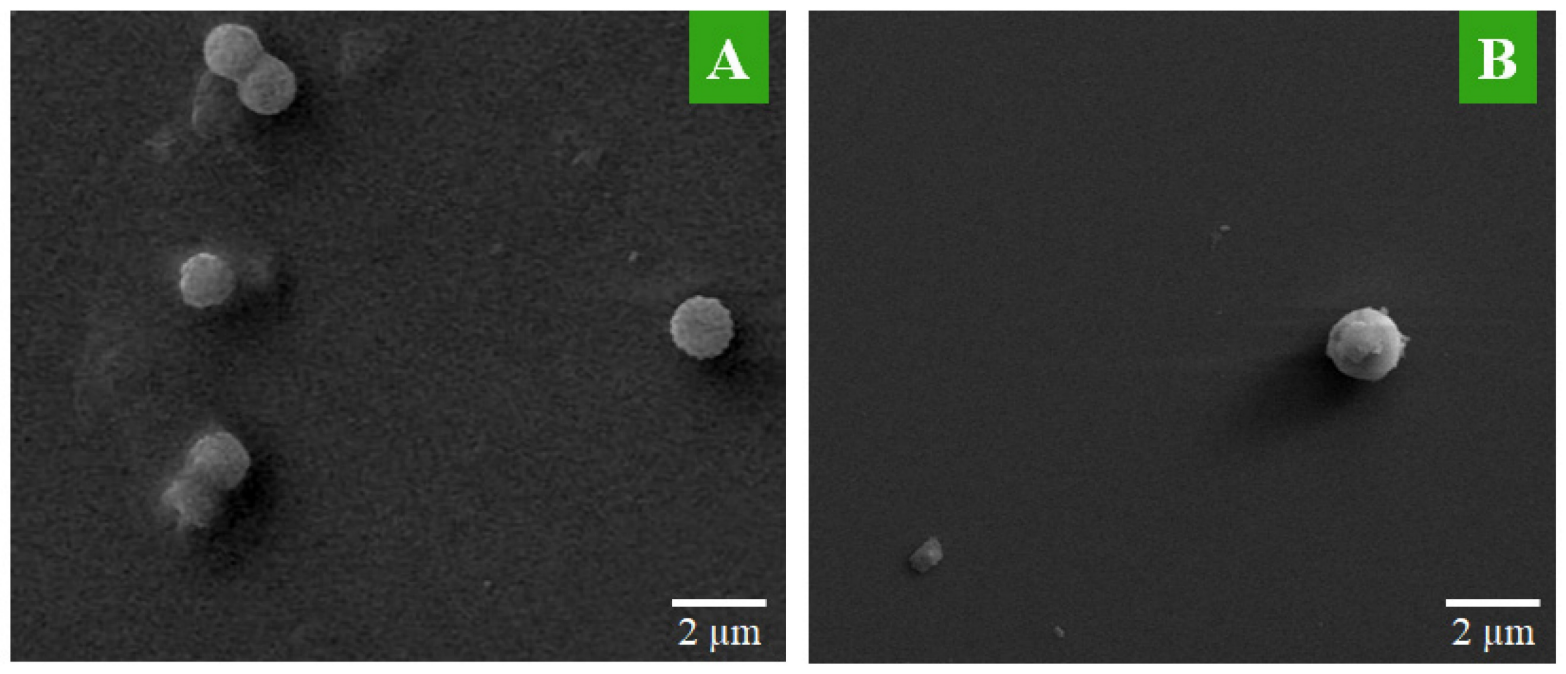
The SEM image of the nanohybrid system(A) MTX-NLS and (B) MTX-NLPHS.

SEM is used to see the surface topography of MTX-NLS and MTX-NLPHS. The spherical structure was seen in the case of MTX-NLS, showing the successful nanocarrier formulation using a single emulsion solvent evaporation method. The spherical structures of MTX-NLS were smooth and had a diameter of approximately 2 μm **(Fig. 3A)**, however, the SEM image of MTX-NLPHS showed a spherical shape with a relatively bigger size **(Fig. 3B).** According to the aforementioned findings, the polymer's long chain may organize into its compact shape. As a result, the lipid monomer surrounding the polymer is consistently aligned and has the right shape with a relatively bigger size. This also means that the PLGA was successfully blended with the lipid system.

### Fourier-transform infrared (FTIR) characterization of the hybrid system

Infrared spectroscopy analysis was conducted to check the characteristics peaks of functionalities of MTX, PC, and PLGA and also used to validate the lipid-PLGA interactions in the final system.

**Figure 4:**
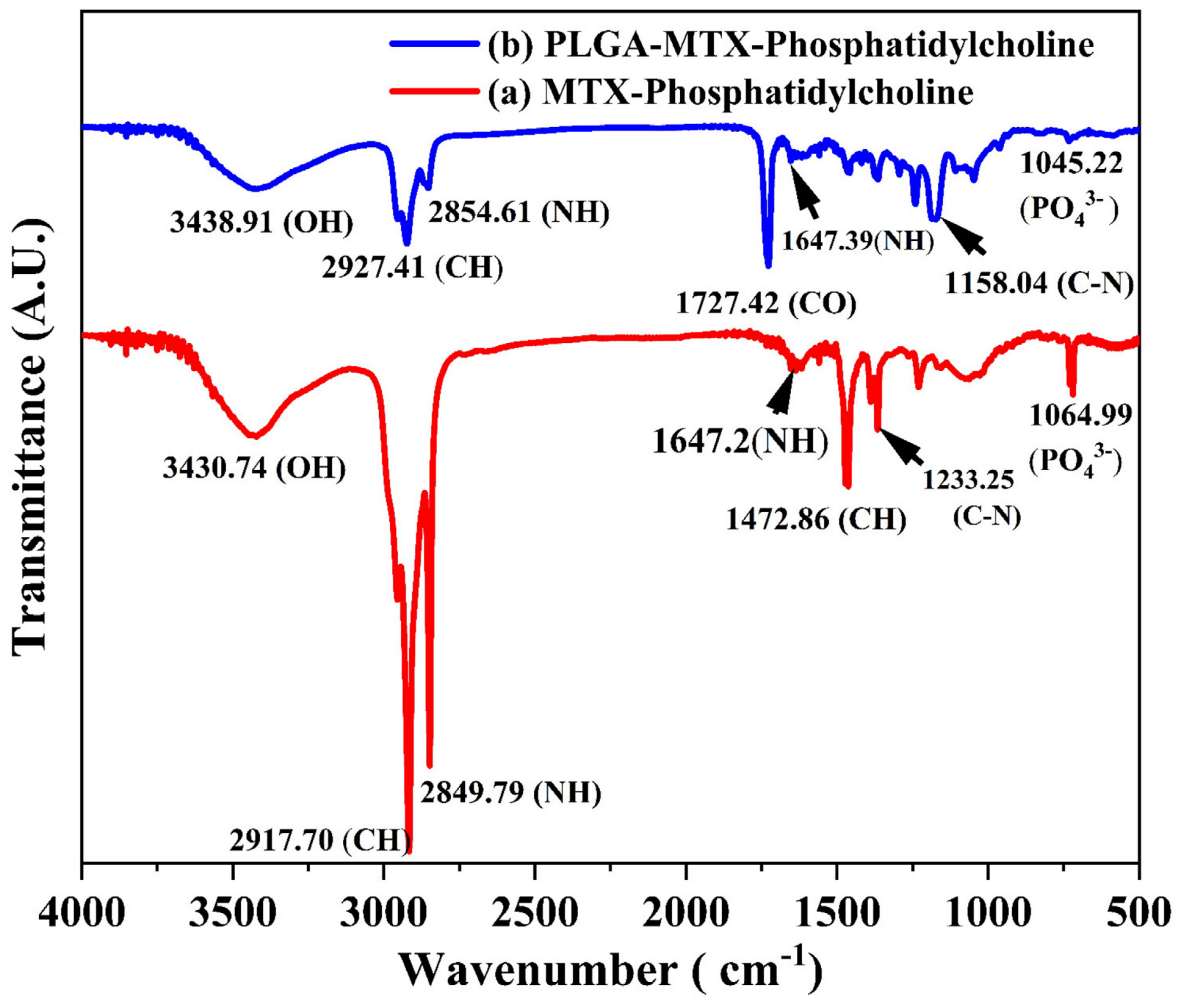
The infrared spectrum of MTX-NLS (red curve) and MTX-NLPHS (blue curve) at the resolution of 4 cm^-1^.

The FTIR spectra of MTX-NLS (red curve) and MTX-NLPHS (blue curve) showed numerous peaks in accordance with various functionalities **(Fig. 4)**. Numerous peaks at wave numbers 3430.71 cm^-1^, 2917.70 cm^-1^, 2849.79 cm^-1^, 1647.2 cm^-1^, 1472.6 cm^-1^, 1233.25 cm^-1^, and 1064.99 cm^-1^, which correspond to OH, CH_2_, NH (stretching), NH (Bending), CH, CN, and PO_4_^-3^, are shown in the red spectrum (MTX-NLS). These peaks were seen because the formulated system contained MTX and lipids. The spectra in the blue curve (MTX-NLPHS) maintain all recognizable peaks with the shifted band. Interestingly, in this spectral region, a new peak was observed at a wavenumber of 1727.42 cm^-1^ which corresponds to the CO group. This signature peak is attributed to the presence of PLGA in the hybrid system. The unique peaks shifted to a higher wavenumber due to non-covalent interactions between PLGA and phosphatidylcholine. These comparisons show that the MTX-NLPHS was successfully formed.

### X-ray diffraction (XRD) analysis

**Figure 5:**
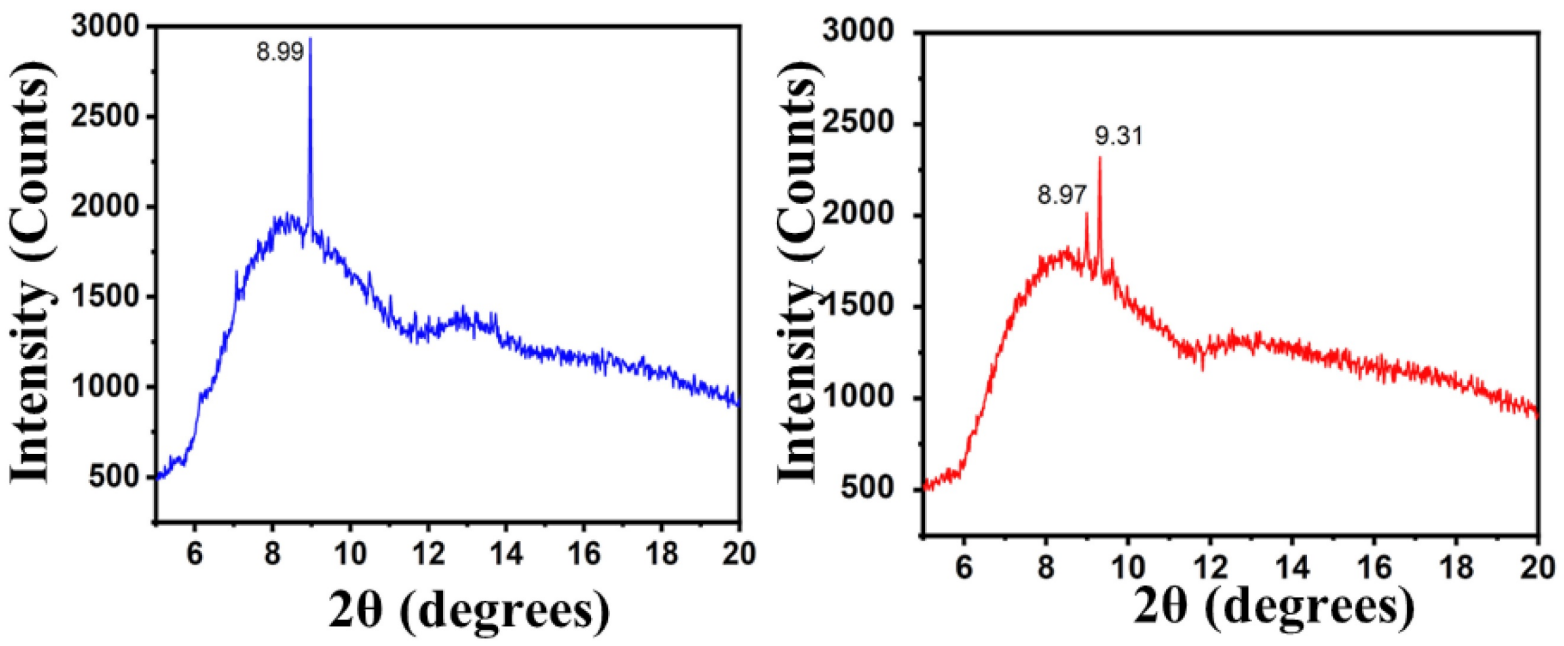
Diffraction pattern of MTX-NLS (blue curve) and MTX-NLPHS (red curve) scanned between 2θ = 5° to 2θ = 20°.

The MTX-NLS and MTX-NLPHS were characterized using XRD to see the crystallinity pattern of the final system after incorporation of the PLGA **(Fig. 5)**. The diffractogram of MTX-NLS showed a characteristic peak at 2θ = 8.99°, which was attributed due to the presence of MTX. In the blue spectral region, a broader peak was observed which was due to the amorphous structure of the lipid. The diffraction peaks of the amorphous structure were scattered due to the absence of absolute positive and destructive X-ray interference in a finite-sized lattice. Interestingly, in the red spectra (MTX-NLPHS), the two characteristic peaks were observed which correspond to 8.97° and 9.31°. The new peak in the XRD pattern of the final system suggests that the lipid bilayer organizes and shifts the structure to the more crystalline structure in the presence of PLGA. In this system, the monomer PLGA may form the long chain of polymer inside the lipid nanocarrier. The characterization above clearly indicates that the successful formation of the MTX-NLPHS can be employed for further applications.

### *In vitro* drug release kinetics

**Figure 6:**
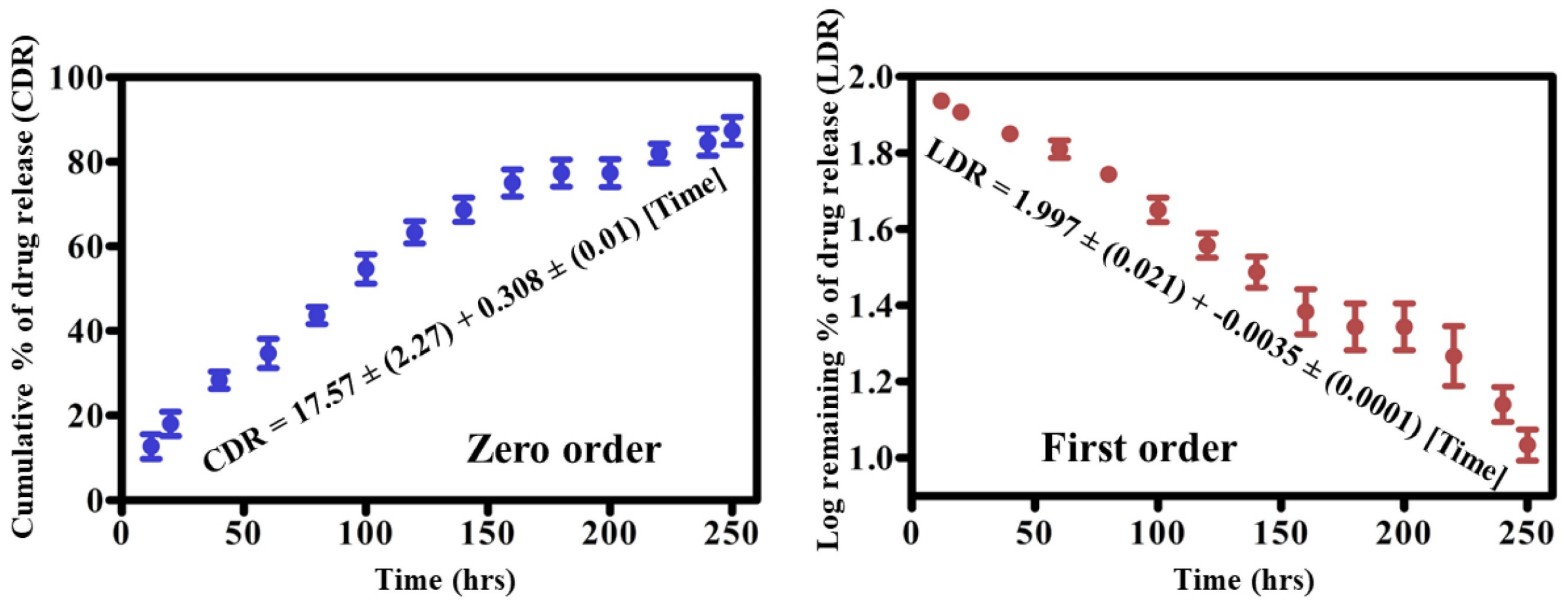
Drug release kinetics of MTX from NLPHS (*In vitro*) in phosphate buffer saline for Zero-order kinetics (blue curve) and first-order kinetics (red curve) at physiological pH with a significance level of (p≤0.05*).

We subsequently studied the drug dissociation in PBS after characterizing the nanohybrid system and studying the drug encapsulation. As shown in **Fig. 6**, the drug dissociation pattern from the formulated nanohybrid system was examined for 250 h. Drug release from the constructed nanohybrid system was explained using zero-order and first-order kinetic models. The kinetic pattern depicted that the 18% drug was released within 20 h. The early fast rate of drug release could be attributed to some drugs being attached to the surface of the lipid. Furthermore, 200 h were required to release 71% of the drugs, and the cumulative percent of drug release (CDR) reached 90% in the 240 h that followed. The slow rate of drug release may ascribe to the presence of PLGA in the core of lipids. This system is helpful in suppressing the growth of cancer cells in a short time. In addition, a restricted or sustained rate of drug release from the constructed hybrid system may favor prolonged circulation of drugs in the blood circulatory system and also overcome the problem of multiple drug resistance in diverse biological applications. Therefore, our system met the requirement of a restricted and sustained rate of drug release after 20 h of incubation at the physiological pH. The constructive drug release pattern between zero and the first-order kinetic pattern was examined using a linear regression equation. Effective CDR is presented as the regression equation for the zero-order curve i.e., CDR= 17.57 ± (2.27) + -0.308 ± (0.01) [Time], with a R^2^ of 0.921. However, the effective CDR for the first order curve is represented as follows: 1.997 ± (0.021) + -0.0035 ± (0.0001) [Time] with an R^2^ of 0.930. The relatively higher R^2^ value for the first order model revealed that the hybrid nanocarrier released the drugs in a concentration-dependent phenomenon.

### Analysis of cell viability

**Figure 7:**
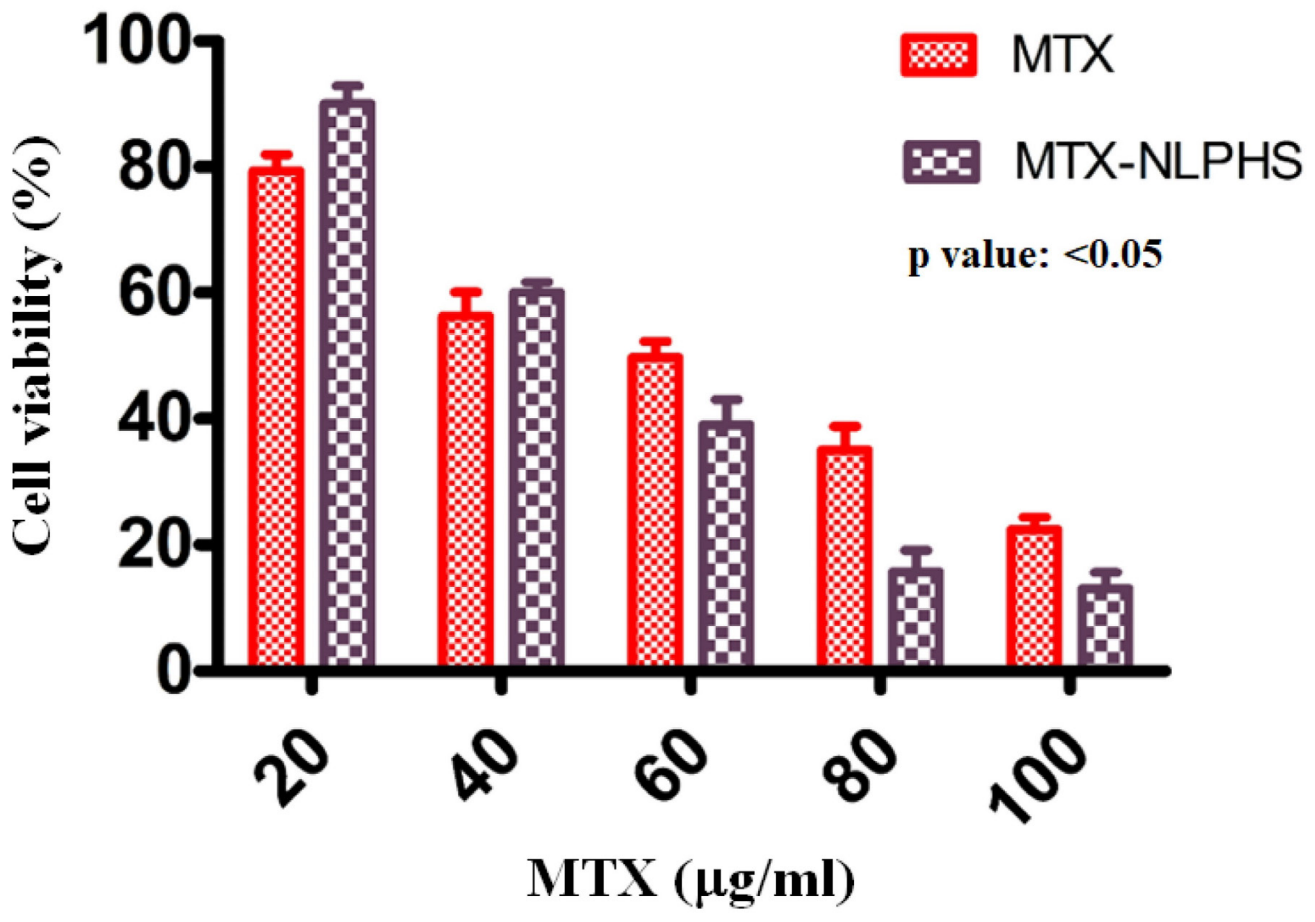
Cell toxicity studies on MCF-7 cells (*In vitro*) treated with inducers MTX and MTX-NLPHS after 48 h of incubation. Values are reported as (mean ± standard error) and significance level as p<0.05.

To assess the cytotoxicity of MTX and MTX-NLPHS on cells, the MTT assay was utilized (**Fig. 7**). MTX was used as a control (red bar) to compare with the formulated sample. The viability of the cancer cells was found to be 79.33 ± 3.8 %, 56.33 ± 5.7 %, 49.67 ± 4.6 %, 35 ± 5.9 %, and 22.33 ± 2.8 % for MTX at 20, 40, 60, 80, and 100 µg /mL, respectively. In another setup, the effect of MTX-NLPHS was examined on the cancer cells (black bars), where the percent viability was determined to be 90 ± 4.8 %, 60 ± 1.9 %, 39 ± 6.1 %, 15 ± 5.8 %, and 11 ± 2.7 % at 20, 40, 60, 80, and 100 µg /mL, respectively. The result suggested that the MTX reduced the cell viability following an increasing concentration of drugs. In this case, cells may uptake the drugs through passive diffusion and subsequently enhance cell toxicity. However, at the lower concentrations of MTX, MTX-NLPHS demonstrated considerably lower cell toxicity, although the toxicity increased with higher concentrations of MTX in the system. The result indicated that at lower concentrations of MTX in the system, the incorporation of PLGA may enhance its stability and control the drug release into the medium which thereby culminate to increase in the viability of the cells. Upon increasing the concentration of MTX in the system, the drug might be released from the carrier thus, disrupting its structural integrity and increasing cell death. The aforesaid findings suggested that the PLGA is a better carrier for the accumulation of MTX at optimum concentration within cells in a controlled manner. The IC50 value for the MTX-NLPHS system was found to be 51.78 µg /mL.

### Intracellular ROS monitoring

**Figure 8:**
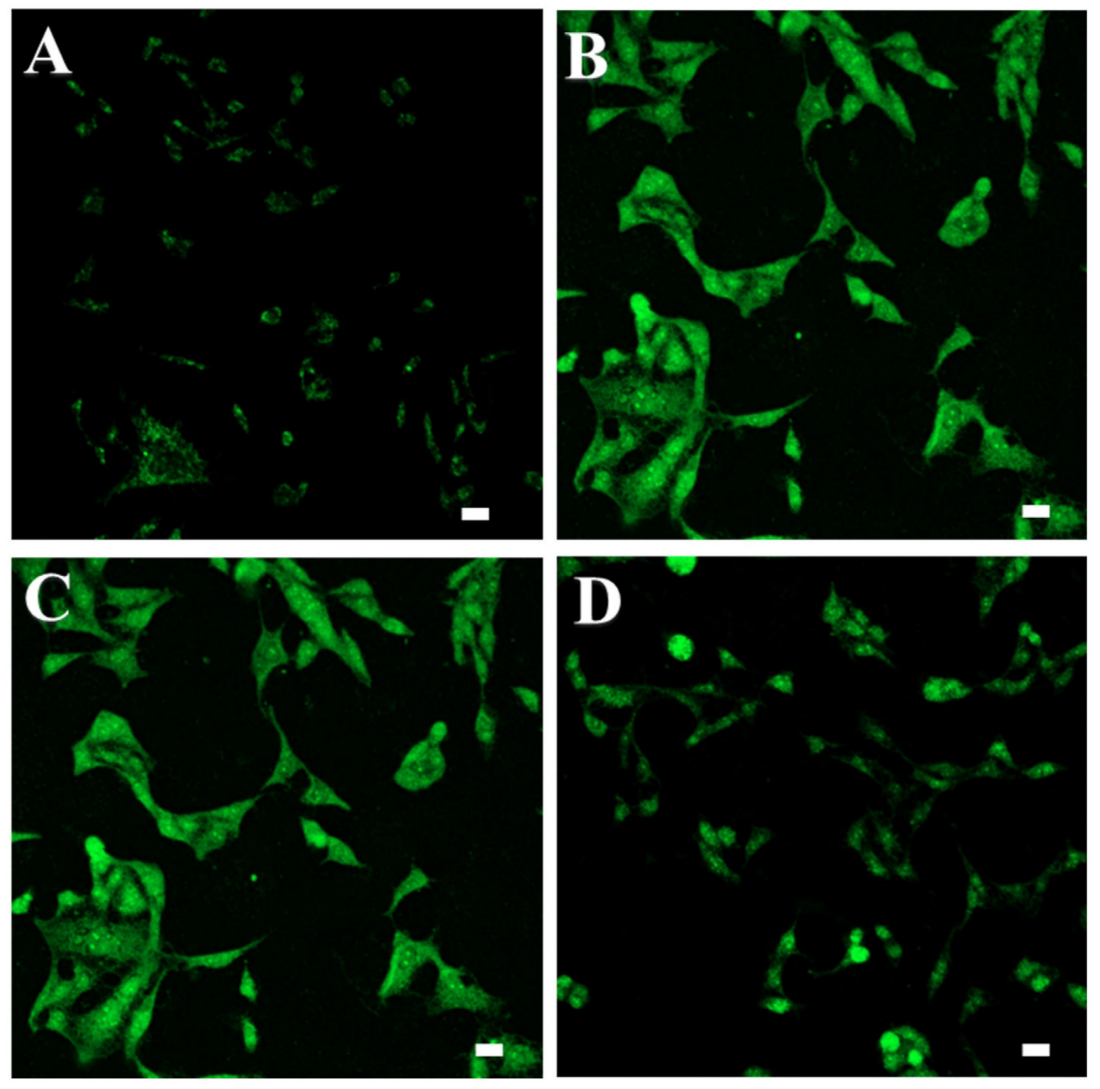
ROS study in MCF-7 cells (A) Untreated cells (Negative control) (B) Cells treated with H_2_O_2_ (Positive control) (C) MTX (D) MTX-NLPHS.

The fluorescent probe DCFDA was used to measure intracellular ROS levels in cells treated with MTX and MTX-NLPHS (**Fig. 8**). Exposure of MCF-7 cells to 50 μM H_2_O_2_ solution significantly increased the formation of ROS which can be represented in terms of mean fluorescent intensity which was found to be 12 a.u. (**Fig. 8B**) compared to untreated cells for which mean fluorescent intensity was found to be 1.39 a.u. (**Fig. 8A**). Treatment of cells with MTX significantly attenuated the ROS formation which can be indicated in terms of mean fluorescent intensity of 11 a.u. (**Fig. 8C**). Further, more attenuation in ROS formation can be seen in the cells exposed to MTX-NLPHS indicated by mean fluorescence intensity of 3.80 a.u. (**Fig. 8D**) which infers more accumulation of drugs inside the cells through hybrid nanocarriers. These results further potentiated the selection of PLGA as a better carrier for controlled transport of drugs into the cells by endocytosis.

### Morphological analysis of MCF-7

**Figure 9:**
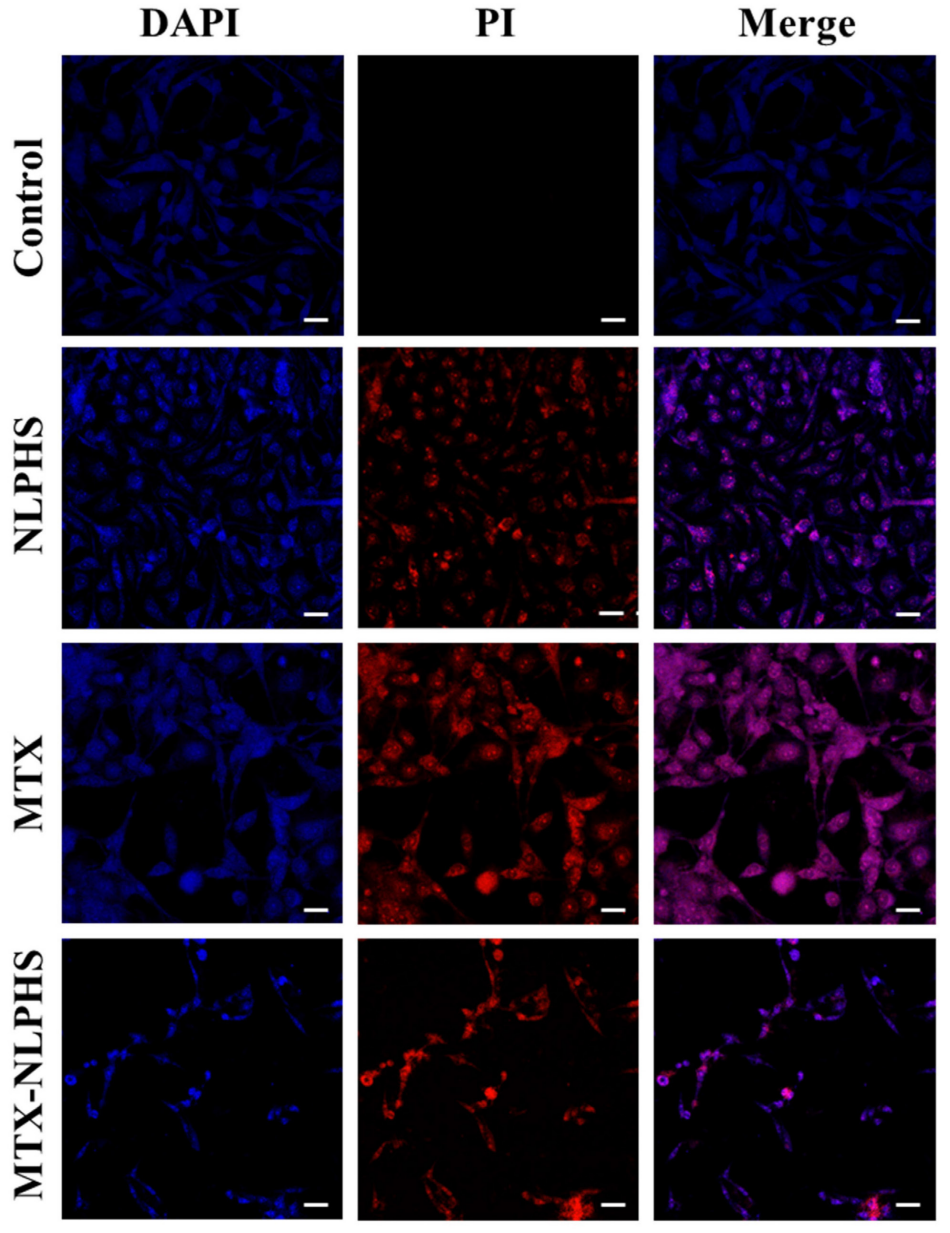
Study of nuclear morphology of MCF-7 cells stained with DAPI and PI dyes using confocal microscopy. Row 1 represents the untreated cells (control), Row 2 represents the cells treated with the hollow NLPHS carrier, Row 3 represents the free MTX-treated cells, and Row 4 represents the MTX-NLPHS-treated cells. Column 1 shows blue fluorescence from DAPI staining, column 2 shows red fluorescence from PI staining, and Column 3 represents the merged images.

The morphological changes induced by MTX and MTX-NLPHS were analysed with DAPI and PI staining using confocal microscopy. DAPI and PI stained cells when exposed to MTX-NLPHS cause higher nuclear elongation, cell shrinkage, and disintegration as compared to MTX-treated and untreated cells** (Fig. 9)**. This finding suggests that cell fusion followed by carrier-mediated endocytosis is presumably the mechanism behind the cellular basis for the internalization of MTX-NLPHS 29. Considering these findings, we can strongly suggest the MTX-NLPHS as a superior drug delivery carrier, particularly in MCF-7 cells that increases the bioavailability of the drugs as compared to free MTX.

## Conclusion

In the experimental work, we have designed NLPHS for MTX drug delivery in MCF-7 cells. Phosphatidylcholine blended with PLGA works as a scaffolding material in which MTX is incorporated for controlled drug distribution. The hybrid system was characterized using SEM, FTIR, XRD, and DLS and showed uniform size with an irregular shape, various functionalities, crystallinity, and chemical stability. The release kinetics pattern of the MTX-NLPHS depicts the controlled delivery of MTX and the system may overcome the problem of multiple drug resistance. Cell culture-based tests such as MTT assay and ROS monitoring analysis confirm the effect of NLPHS on the cellular system. Further, confocal microscopy suggests that the cell morphology significantly changed after treatment with MTX-NLPHS, which again potentiates the use of NLPHS as a better carrier for controlled drug delivery into the cells. Moreover, *in vivo* tests will be conducted to check the efficacy of the constructed MTX-NLPHS.

## Figures and Tables

**Figure 1:**
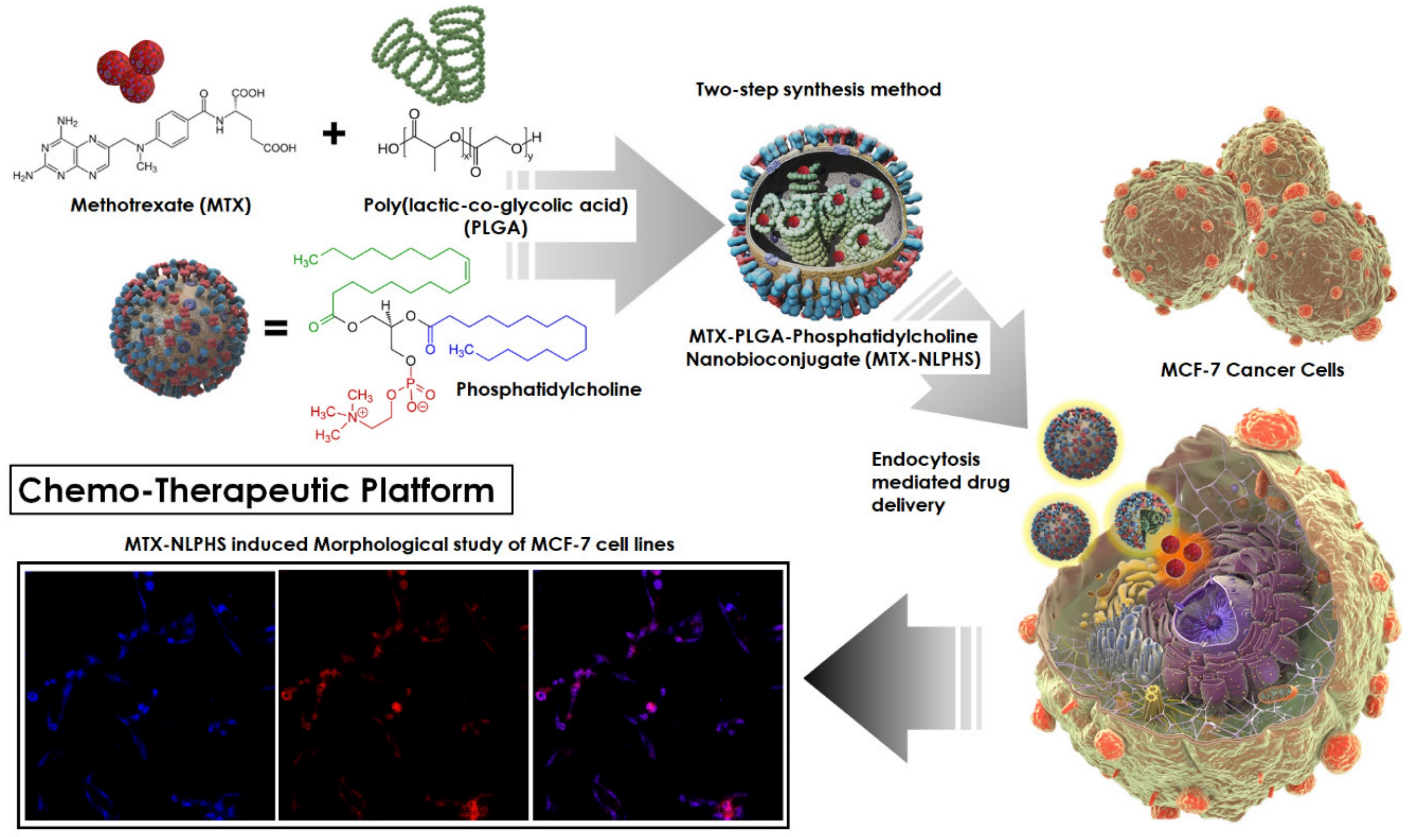
The figure depicting the schematic representation of nanohybrid system formulation and its cellular internalization.

**Table 1:** Exhibits the particle size, polydispersity, zeta potential, and encapsulation efficiency of MTX-NLS and MTX-NLPHS

Sample	Particle size (nm)	Polydispersity	Zeta potential (mV)	Encapsulated efficiency (%)
MTX-NLS	102 ± 4.67	0.11 ± 0.040	-34 ± 2.51	79.40 ± 0.28
MTX-NLPHS	198 ± 8.44	0.134 ± 0.048	-28 ± 3.50	86.48 ± 0.31
